# The effects of family environment cognition and its difference perceived by adolescents and their parents on the treatment effect of non-suicidal self-injury behaviors in adolescents: a 1-year prospective cohort study

**DOI:** 10.3389/fpsyt.2023.1183916

**Published:** 2023-09-12

**Authors:** Yalan Li, Xin Li, Yunge Li, Yu Xiao, Chunya Li, Jia Chen, Yao Li, Lishi Luo, Ding Su, Juan Jia, Haofei Cheng, Tianjiao Liu, Na Du

**Affiliations:** ^1^The Fourth People’s Hospital of Chengdu, Psychosomatic Medical Center, Chengdu, China; ^2^Chengdu Women’s and Children’s Central Hospital, School of Medicine, University of Electronic Science and Technology of China, Chengdu, China

**Keywords:** non-suicidal self-injury, adolescent, family environment, prospective study, treatment

## Abstract

**Introduction:**

Family environment is the primary environment for adolescent growth and development, which is believed to have an important impact on the occurrence of non-suicidal self-injury (NSSI) behavior in adolescents. This study aimed to explore the effects of family environment cognition and cognitive differences perceived by adolescents and their parents on the treatment effects of NSSI in adolescents and to provide more potential perspectives for NSSI treatment.

**Methods:**

A one-year prospective longitudinal sub-cohort investigation was carried out among 199 adolescents engaged in NSSI and one of their important guardians from the Longitudinal Psychosomatic Disease Study (LoPDS). The NSSI behaviors of adolescents were evaluated at 3 months, 6 months and 1 year after enrollment. The family environment scale (FES) and NSSI Behavior Questionnaire were used as assessment tools for family environment and adolescents NSSI behaviors. Multiple linear regression was used to investigate the role of family environment perception difference in the treatment effect of adolescent NSSI.

**Results:**

After one year of follow-up, the perceived self-injury impulse score in recent 2 weeks, self-injury impulse frequency in recent 2 weeks, total number of self-injury in recent 2 weeks decreased significantly. The higher the adolescent family cohesion (Beta: 1.130, 95% CI: 0.886,1.373; p=0.032), parental family expressiveness (Beta: 0.818, 95% CI: 0.375,1.260; p=0.037) and parental family active-recreational orientation score (Beta: 0.609, 95% CI: 0.236,0.981; p=0.048), the better the treatment effect. However, higher adolescent family conflict (Beta: -0.838, 95% CI: -1.377,-0.298; p=0.024) were associated with lower treatment outcomes. The greater the cognitive difference between parents and adolescents in family cohesion (Beta: -1.307, 95% CI: -2.074,-0.539; p=0.014) and family conflict(Beta: -0.665, 95% CI: -0.919,-0.410; p=0.037), the worse the therapeutic effect of NSSI might be.

**Discussion:**

There were certain differences in the cognition of family relationships between parents and adolescents, and subjective family relationship cognition and cognitive differences had a significant effect on the treatment effect of NSSI in adolescents. Helping them identify the cause of cognitive differences and conducting systematic family therapy from the points of difference may be another perspective to improve the treatment effect of NSSI in adolescents.

## 1. Introduction

Non-suicidal self-injury (NSSI) is characterised by intentional, direct, and repeated injury or destruction of one’s own body tissues without suicidal intention, and its incidence rate in adolescence is reported to be between 7.3 and 17.2% (1–3). The incidence rate of NSSI is higher among adolescents with psychological disorders than among adolescents without psychological disorders. Data from a mental health center in China showed that from 2016 to 2021, the proportion of mental disorders combined with NSSI increased from 29.2 to 95.9% (4).

The prevalence of NSSI peaks in mid-adolescence (approximately age 15–16 years) and decreases in late adolescence (approximately age 18 years) (5, 6). Although the prevalence of NSSI is significantly reduced in late adolescence, adolescents with repetitive NSSI appear to have other dysfunctional behaviors even after the treatment of NSSI. A recent study has suggested that adolescents who stop repeating NSSI are likely to exhibit high levels of substance abuse (7). In addition, NSSI is a significant risk factor for suicide attempts and suicides (8, 9). There is an approximately 30-fold higher suicide risk in patients with NSSI compared with the general population (10, 11).

Previously, NSSI was considered a symptom of borderline personality disorder (12). However, recently, most studies tend to consider NSSI an independent mental disorder (13, 14). Current studies have demonstrated that NSSI behaviors are mainly affected by individual psychological, environmental, and neurobiological factors. NSSI behavior is not the result of a single factor but the result of a combination of neurobiological factors related to genetics and acquired factors, such as personality, family, and adverse life events (15–18). This behavior may occur in isolation or in association with several specific psychiatric syndromes. Therefore, there are no unified international treatment guidelines for NSSI. For patients with other mental diseases, a targeted medication for complications is required. However, there are no specific drugs available for the treatment of NSSI. Moreover, different patients have significantly different treatment effects. Improving the therapeutic effect in patients with NSSI is an urgent problem that needs to be solved.

Environmental factors, especially the family environment, are the primary factors for adolescent growth and development, which have an important effect on adolescent mental health and are closely related to adolescent self-awareness, behaviors, and psychological symptoms (19–21). Previous studies have mainly focused on the effects of objective family environment on NSSI in adolescents. However, family relationships are interpersonal relationships between family members. In addition to the objective family environment, family members’ subjective cognition of the family environment is also important (22, 23). Moreover, our previous study found significant differences between parents and adolescents in their cognition of the familyenvironment.

Thus, we conducted a 1 year prospective cohort study on patients with NSSI. This study collected the family environment cognition data of adolescents and their parents and the treatment effects of NSSI. This study aimed to explore the effects of family environment cognition and cognitive differences perceived by adolescents and their parents on the treatment effects of NSSI in adolescents and to provide more potential perspectives for NSSI treatment.

## 2. Methods

### 2.1. Study design and participants

The present study was embedded in the Longitudinal Psychosomatic Disease Study (LoPDS), an ongoing psychosomatic disease cohort study in adolescents conducted in Chengdu that aims to determine the relative contributions of biological factors and the environment to the development and prognosis of psychosomatic diseases (Chinese Clinical Trial Registry ChiCTR2200059437). This study was approved by the Ethics Committee of the Fourth People’s Hospital of Chengdu (No. 201830). This prospective sub-cohort study was conducted at the Fourth People’s Hospital of Chengdu and recruited all adolescents who engaged in NSSI behaviors between January 2019 and December 2020. Written informed consent was obtained from all participants and their parents or guardians. Patients with generalised developmental disorders, intellectual disability, schizophrenia pedigree, drug abuse, or other severe somatic diseases were excluded to reduce confusion.

Patients aged <12 or > 18 years were not included in this study. Those who were unable to participate in follow-ups and were unable to conduct the standardised questionnaire assessment were considered to have dropped out. Simultaneously, we invited one of the adolescents’ parents/guardians to complete the questionnaires at baseline. All adolescents needed to complete the subsequent three follow-ups at 3 months, 6 months, and 1 year thereafter. At each follow-up visit, patients were interviewed in the outpatient clinic or by telephone to determine their current condition. A total of 350 adolescents with NSSI behaviors as part of the LoPDS study were initially recruited for this subgroup study. After excluding parent–adolescent pairs who did not meet the inclusion criteria or did not complete a standardised questionnaire assessment, suicide, or were unable to participate in follow-up, the final analysis included 199 parent–adolescent pairs with NSSI, which can meet the quantity for statistical analysis.

### 2.2. Data collection

Standardised questionnaires (Supplementary File 1) were used to collect the sociodemographic data of participants (including sex, age, educational background, parents’ marital status, parents’ educational background, parents’ age, and family life structure).

The Family Environment Scale-Chinese Version (FES-CV) (Supplementary File 2) was used to collect the perception of the participants’ family relationships at baseline. It can reflect nine aspects of family relationship, including family cohesion, expressiveness, conflict, independence, achievement orientation, intellectual–cultural orientation, active–recreational orientation, moral–religious emphasis, organization, and control (24, 25). The FES-CV was revised and rewritten by Fei Lipeng et al. in 1991 based on the Family Environment Scale (FES) prepared by Moss, an American psychologist, which has acceptable internal consistency and retest reliability in the Chinese population (26). Cognitive differences in family relationships between adolescents and their parents were defined as the differences between the scores of each item of the parents’ FES-CV and the scores of adolescents’ FES-CV.

The NSSI behavior questionnaire (Supplementary File 3) was used to evaluate the NSSI behaviors of participants at the time of enrolment, after 3 months treatment, 6 months treatment, and 1 year treatment. This questionnaire contains three parts: the first part is the participant’s self-assessment of the impulse to self-harm in the past 2 weeks, selected according to “0–10 points”, with the degree assessed from nothing to strong. The second part assesses the total number of self-injuries in the last 2 weeks, including self-injury behaviors without evident tissue injury and self-injury behaviors with significant tissue damage. The third part assesses whether the participant has had the urge to self-harm and the frequency of self-injury impulse in the past 2 weeks and uses a 7-level score to classify and select according to the frequency of self-injury impulse: grade 1 is once every 2 weeks; grade 2, once a week; grade 3, twice a week; grade 4, more than thrice a week; grade 5, more than five times a week; grade 6, an average of one time per day; and grade 7, an average of more than two times a day. Within a year, there were a total of 4 follow-up visits: at the time of enrolment, after 3 months treatment, 6 months treatment, and 1 year treatment.

### 2.3. Diagnostic criteria of non-suicidal self-injury

According to the 5th Edition of the Diagnostic and Statistical Manual of Mental Disorders (27, 28), NSSI was diagnosed based on the description that the individual has, on 5 or more days, engaged in intentional self-inflicted damage to the surface of his or her body in the last year, of a sort likely to induce bleeding, bruising, or pain (e.g., cutting, burning, stabbing, hitting, and excessive rubbing), for purposes not socially sanctioned (e.g., body piercing and tattooing), but performed with the expectation that the injury will lead to only minor or moderate physical harm.

### 2.4. Treatment process of NSSI

Recently, there are no unified international treatment guidelines for NSSI. At our hospital, the commonly used therapeutic methods in clinical practice are mainly divided into psychological, drug, and physical therapies (29–31). In our cohort, all patients were treated using a combination of medical, physical, and psychological approaches after enrolment by psychiatrists or medical technicians who have received professional training. All participants were administered Food and Drug Administration-approved antidepressants (sertraline/fluoxetine) for the treatment of anxiety and depression and mood stabilisers (lithium carbonate or valproate) to stabilise their mood. Group psychotherapy and repeated transcranial magnetic stimulation therapy were administered 5 times a week after enrolment during the first month of treatment and after 1 month, with medication maintenance therapy.

### 2.5. Statistical analyses

All statistical analyses were performed using the Statistical Package for the Social Sciences version 25.0 (IBM Corp., Armonk, NY, United States). The chi-squared or Fisher’s exact test was used to assess categorical data, which were reported as counts and percentages. The means and standard deviations of continuous variables were calculated using Student’s *t*-test, least significant difference Student’s *t*-test, one-way analysis of variance, or non-parametric test. Multiple linear regression analysis was performed to explore the association between the treatment effects of NSSI and parental/adolescent family environment cognition or the difference between them. Covariates were selected according to the significantly different variables in the univariate analysis and factors reported in previous studies that would affect the dependent variable. All tests were two-tailed, and statistical significance was set at *p* < 0.05.

## 3. Results

**Figure 1:**
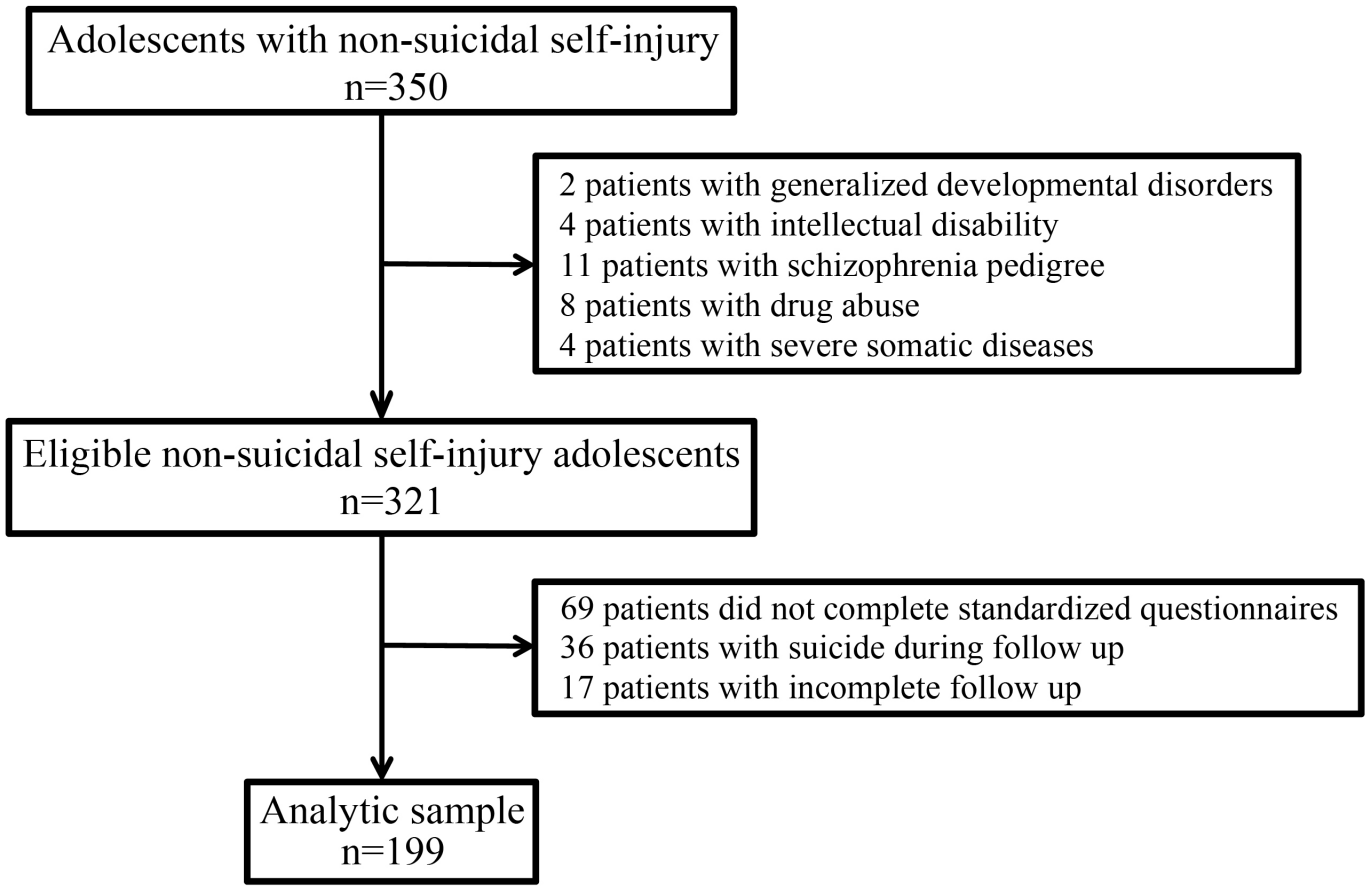
The selection process for this study population.

The selection process of the study population is shown in Figure 1. A total of 350 adolescents with NSSI behaviors as part of the LoPDS study were initially recruited for this subgroup study. After excluding parent–adolescent pairs who did not meet the inclusion criteria or did not complete a standardised questionnaire assessment, suicide, or were unable to participate in follow-up, the final analysis included 199 parent–adolescent pairs with NSSI. The descriptive data of the study participants are shown in Table 1. The average age at baseline was 15.44 ± 1.90 years, 87.9% of the participants were female, and 58.3% lived in the city. Moreover, 94.5 of the participants were still at school, and 30.1% of their parents had been divorced or remarried. Approximately half of the adolescents were left-behind children, and 10.9% had a family history of psychosis. The main diagnoses of the participants were as follows: depressive episode (43.5%), anxiety disorder (16.9%), post-traumatic stress disorder (9.0%), bipolar disorder (8.0%), personality disorder (7.0%), and obsessive-compulsive disorder (14.6%).

**Table 1:** Description of the participants’ characteristics.

Variables	Total
Number	199
Age (year)	15.44 ± 1.95
Sex (female)	175 (87.9%)
Nationality (Han)	189 (95.0%)
Habitation (city)	116 (58.3%)
Education	
Primary school	7 (3.5%)
Junior high school	85 (42.7%)
High school	83 (41.7%)
University	24 (11.1%)
Working conditions	
Students	188 (94.5%)
Stop schooling	11 (5.5%)
Have family history of psychosis	21 (11.1%)
Marital status of parents	
In marriage	139 (69.8%)
Divorce	42 (21.1%)
Remarriage	18 (9.0%)
Left-behind children	98 (49.2%)
Family structure	
With parents	110 (55.3%)
With one of parents	53 (26.6%)
With non-parents	36 (18.1%)
Diagnosis	
Depressive disorder	88 (43.5%)
Anxiety disorder	34 (16.9%)
Bipolar disorder	16 (8.0%)
Post-traumatic stress disorder	18 (9.0%)
Personality Disorder	14 (7.0%)
Obsessive-compulsive disorder	29 (14.6%)

During treatment, the patients in the cohort were continuously followed up at baseline, after 3 months treatment, 6 months treatment, and 1 year treatment. The perceived self-injury impulse score in the last 2 weeks, self-injury impulse frequency in the last 2 weeks, and total number of self-injuries in the last 2 weeks were used to assess the therapeutic effect, which all dropped significantly (*p* < 0.001) (Table 2).

**Table 2:** Score trajectory of NSSI scales during follow-up.

Variables	At baseline	3 month treatment	6 month treatment	1 year treatment	*p*-value
Perceived self injury impulse score in recent 2 weeks	7.50 ± 2.48	4.92 ± 2.29	3.93 ± 2.49	2.90 ± 2.63	<0.001^a^
Self injury impulse frequency in recent 2 weeks	3.99 ± 2.28	3.18 ± 1.70	2.58 ± 1.74	2.01 ± 1.90	<0.001^a^
Total number of self-injury in recent 2 weeks	27.65 ± 40.04	12.29 ± 19.01	8.67 ± 13.93	6.46 ± 12.18	<0.001^a^

NSSI, non-suicidal self-injury.

a Average and standard deviation. Welch’s *t*-test.

Further comparing the subjective perception differences in family relationships between parents and adolescents, the results showed that adolescents with NSSI rated positive family relationships (family cohesion, expressiveness, independence, active–recreational orientation, moral–religious emphasis, and organization) lower than their parents, but they had a higher perception of family conflict than their parents (*p* < 0.05). There was no significant difference in parent–adolescent’s cognition of family achievement orientation and intellectual–cultural orientation (Table 3).

**Table 3:** The differences in the perception of family relationship between adolescents and parents.

Variables	Parents	Adolescents	*p*-value
Family			
Cohesion	4.81 ± 2.59	3.70 ± 2.51	<0.001^a^
Expressiveness	4.18 ± 1.63	3.49 ± 1.75	<0.001^a^
Conflict	4.21 ± 2.47	5.61 ± 2.15	<0.001^a^
Independence	5.37 ± 1.63	4.91 ± 1.53	0.004^a^
Achievement Orientation	5.18 ± 1.84	4.95 ± 1.72	0.217^a^
Intellectual-Cultural Orientation	3.13 ± 1.90	3.22 ± 1.77	0.643^a^
Active-Recreational Orientation	3.64 ± 2.19	3.15 ± 2.17	0.025^a^
Moral-Religious Emphasis	5.05 ± 1.80	4.63 ± 1.55	0.014^a^
Organization	5.26 ± 2.00	4.43 ± 2.06	<0.001^a^
Control	3.38 ± 1.77	3.61 ± 2.10	0.236^a^

a Average and standard deviation. Student’s *t*-test.

Although patients with NSSI have evident therapeutic effects in general, there are significant differences among individuals. Therefore, the 2 weeks total number of self-injury differences between the 1 year treatment and baseline was used as the evaluation standard of the treatment effect to further analyse the influence of family environment factors on the treatment effect of patients with NSSI. After adjusting for age, sex, family history of psychosis, left-behind children, and partial content of FES, the results revealed correlations between the treatment effect of NSSI and adolescent family cohesion, adolescent family conflict, parental family expressiveness, and parental family active–recreational orientation. The higher the adolescent family cohesion (beta, 1.130; 95% confidence interval [CI], 0.886–1.373; *p* = 0.032), parental family expressiveness (beta, 0.818; 95% CI, 0.375–1.260; *p* = 0.037), and parental family active–recreational orientation score (beta, 0.609; 95% CI, 0.236–0.981; *p* = 0.048), the better the treatment effect. However, higher adolescent family conflict (beta, −0.838; 95% CI, −1.377 to −0.298; *p* = 0.024) was associated with lower treatment outcomes (Table 4).

**Table 4:** Association between the parental and adolescent family cognition and treatment effect of NSSI.

Variables	Beta	95% CI	*p*-value	VIF
Age (year)	−0.076	(−1.154,1.003)	0.890	1.143
Sex (female)	2.747	(−3.563,9.057)	0.392	1.093
Have family history of psychosis	1.273	(−2.778,5.324)	0.536	1.062
Left-behind children	0.459	(−6.077,6.996)	0.890	1.088
Adolescent’s cognition				
Family cohesion	1.130	(0.886,1.373)	0.032	1.568
Family expressiveness	−0.647	(−1.983,0.689)	0.341	1.411
Family conflict	−0.838	(−1.377,−0.298)	0.024	1.382
Family independence	0.375	(−0.945,1.696)	0.576	1.058
Family active-recreational orientation	−0.198	(−1.322,0.926)	0.729	1.531
Family moral-religious emphasis	0.364	(−0.977,1.705)	0.593	1.115
Family organization	0.518	(−0.792,1.828)	0.436	1.384
Parents cognition				
Family cohesion	−0.126	(−1.266,1.015)	0.828	1.516
Family expressiveness	0.818	(0.375,1.260)	0.037	1.505
Family conflict	−1.032	(−2.094,0.031)	0.057	1.820
Family independence	−1.251	(−2.605,0.103)	0.070	1.289
Family active-recreational orientation	0.609	(0.236,0.981)	0.048	1.495
Family moral-religious emphasis	−0.425	(−1.579,0.729)	0.469	1.145
Family organization	0.311	(−0.857,1.478)	0.600	1.302

Considering the significant differences between parents and adolescents in their subjective perceptions of family relationships, multiple linear regression analysis was performed to analyse the effects of parent–adolescent cognitive differences in family relations (parent score − teenager score) on the therapeutic effects of NSSI. After adjusting for age, sex, family history of psychosis, and left-behind children, parent–adolescent perceptions of family cohesion and family conflict were significantly related to the treatment effect. Differences between parents and adolescents in family cohesion (beta, −1.307; 95% CI, −2.074 to −0.539; *p* = 0.014) and family conflict (beta, −0.665; 95% CI, −0.919 to −0.410; *p* = 0.037) would reduce the treatment effect. The greater the cognitive difference between parents and adolescents in family cohesion and conflict, the worse the therapeutic effect of NSSI (Table 5).

**Table 5:** Association between the family cognitive differences of parents and adolescents and treatment effect of NSSI.

Variables	Beta	95% CI	*p*-value	VIF
Age (year)	−0.147	(−1.196,0.902)	0.783	1.111
Sex (female)	2.665	(−3.542,8.872)	0.398	1.086
Have family history of psychosis	1.766	(−2.259,5.792)	0.388	1.077
Left-behind children	0.034	(−6.392,6.461)	0.992	1.080
Differences between parents and adolescents				
Family cohesion	−1.307	(−2.074,−0.539)	0.014	1.830
Family expressiveness	0.561	(−0.393,1.515)	0.248	1.306
Family conflict	−0.665	(−0.919,−0.410)	0.037	1.478
Family independence	−0.719	(−1.668,0.231)	0.137	1.124
Family active-recreational orientation	−0.272	(−1.146,0.602)	0.540	1.381
Family moral-religious emphasis	−0.521	(−1.484,0.442)	0.287	1.118
Family organization	−0.095	(−1.069,0.878)	0.847	1.306

Binary logistic regression was used to explore the association between the occurrence of suicide and family environmental factors. After adjusting for the family structure and contents of the FES, the results revealed correlations between the occurrence of suicide and age, sex, family history of psychosis, left-behind children, adolescent family conflict, adolescent family independence, and parental family expressiveness. Female sex (odds ratio [OR], 6.921; 95% CI, 2.826–16.945; *p* < 0.001), family history of psychosis (OR, 4.023; 95% CI, 1.791–12.589; *p* = 0.080), and left-behind children (OR, 3.831; 95% CI, 3.013–9.410; *p* = 0.010) increased the risk of suicide by 6.9, 4.0, and 3.8, respectively. Moreover, the risk of suicide was reduced by 27.9% when age increased by 1 year (OR, 0.721; 95% CI, 0.558–0.931; *p* = 0.012). Regarding the association between the occurrence of suicide and parental and adolescent family cognition, the risk of suicide reduced by 26.0 and 37.3% when adolescent family independence and parental family expressiveness increased by 1 score, respectively. However, the risk of suicide increased by 39.6% when adolescent family conflict increased by 1 score (Supplementary Table S1).

## 4. Discussion

In this 1 year prospective preliminary study, we investigated parent–adolescent family relationship cognition data and analysed the influence of cognition and cognitive differences on the treatment effect of NSSI. This study found that adolescents’ perceptions of family cohesion, adolescent family conflict, parental family expressiveness, and parental family active–recreational orientation affected the efficacy of NSSI treatment in adolescents. Differences in adolescent and parental cognition of family cohesion and family conflict were also strongly associated with NSSI outcomes.

In our cohort, the proportion of girls in the test was significantly higher than that of boys. A previous study has demonstrated that the rate of self-mutilation in women is higher than that in men. This may be because adolescent girls are more precocious than boys, and their sex and self-awareness gradually increases. Although the level of intelligence is significantly improved due to changes in hormone levels, the psychological state and emotional regulation ability of some girls will become unstable (32–34). Nearly half of them are left-behind children. An unstable family relationship may lead to more social adjustment problems among adolescents (35, 36). Parents go out to work, resulting in adolescents getting less parental care, easily having a sense of alienation from their parents and forming a negative perception of the unfavourable situation, and then inducing negative emotions and NSSI behaviors. The lack of parental care has a significant positive predictive effect on the behaviors of adolescents with NSSI (37). A complete family structure is conducive to the play of good family functions and is the foundation for the healthy physical and mental development of adolescents.

Consistent with previous studies, this study found that adolescents with NSSI consistently rated the positives in family relationships lower than their parents (38, 39). In other words, adolescents tend to view family relationships as negative. Adolescents reported higher levels of family conflict than parents, whereas their intimacy and emotional expressions were lower than those of their parents (40, 41). They are more likely to be dissatisfied with their families than their parents are. During adolescence, important changes occur in family relations, and there is a sharp increase in intergenerational conflicts or differences between parents and children. The increase in intergenerational conflict affects differences in parents’ and adolescents’ perceptions of family relationships and the quality of parenting. Differences in the perception of family relationships between adolescents and parents may reflect poor family adjustment during adolescent growth. Adolescents tend to strive for greater autonomy and try to renegotiate their family relationships (42). Cognitive changes enable adolescents to question others’ views (43). If parents are unable to perceive and support such development or if adolescents are unable to adapt to such development, they may develop cognitive and emotional dysfunctions, and further maladaptive behaviors, such as NSSI, will occur.

Notably, this study suggests that the higher the scores of adolescents’ perception of family cohesion, the more NSSI behavior decreases after 1 year; the higher the adolescent family conflict, the worse the effect of NSSI treatment. Family cohesion reflects the emotional connection between personal perception and family and is an indicator of family intimacy and a positive family atmosphere (29, 30). Individuals who grow up in unhealthy family relationships cannot reasonably regulate their emotions or take extreme measures (such as NSSI) when facing negative emotions. Individuals with high family cohesion are conducive to the formation of positive, optimistic, and other good psychological traits in the process of growth and are more able to reasonably deal with negative events (44, 45).

Parental positive cognition of family expressiveness and family active–recreational orientation helps adolescents reduce the occurrence of NSSI behaviors, which may be due to parental emotional expression and communication activities for their children, affecting their future behavioral cognition, emotional expression, and emotion regulation (19, 46). In addition, adolescents are in a critical period of independent needs and independent consciousness development, and parents will try to control their children through strong control of the changes in parent–child relationship during this period, reduce family entertainment activities, and try to increase their connection with children. Adolescents experience a sense of contradiction in the intense conflict between autonomous development and parental control, and to get rid of their parents’ shackles and realise the desire for independent exploration, they will see parental control as a risk factor that threatens their independence and even normal intimate relationships as a threat. To further confirm the independence of the self, it will prove itself by considering NSSI behaviors (21, 47). Thus, parents can improve their parent–child relationship through positive emotional expression, which is conducive to enhancing emotional communication with children, thus improving the closeness between parents and children, forming a virtuous circle of parent–child relationships, and helping to cultivate children’s good behaviors. Simultaneously, parents reduce the control over adolescents, provide children more power to make independent choices, allow adolescents to express different views, and accept children’s negative emotions, which may be helpful in preventing and reducing the occurrence of NSSI behaviors.

Cognitive differences between parents and children regarding family relationships were mainly concentrated in the evaluation of family cohesion and conflict, which significantly affected the treatment effect of adolescent NSSI behaviors (48–50). When a child has already committed self-harm, if there is a lack of emotional resonance between parents and children and there is no sense of family intimacy or family conflict, parents cannot truly understand and accept the child’s emotional changes and self-harming behaviors, increasing family conflict. An increase in family conflict may, in turn, lead to the frequent occurrence of NSSI and poor treatment effects. According to the strain theory, when an individual encounters a certain crisis situation, is at a certain pressure for a long time, and does not have sufficient ability to cope, he/she will experience coping with torque, and the extreme torque consequence is self-harm or even suicide (51–53). When parents and children have the same feelings about the family relationship, both parties believe that family cohesion is higher and family conflict is less, reflecting a warm and safe positive parent–child relationship, which is a protective factor for bad behaviors. If both parties feel a low degree of family cohesion and family conflicts, adequate family support and social support need to be provided during the treatment process, and psychological intervention can be actively provided, which can effectively improve the occurrence of NSSI behaviors in adolescents (36, 54, 55).

The strengths of our study are the specialised study population and large sample size. The study participants were screened using the inclusion and exclusion criteria. Patients with generalised developmental disorders, mental disorders, schizophrenia pedigree, or other psychotic disorders were excluded to reduce confusion. Additionally, the effect of NSSI treatment in adolescents was tracked throughout the 1 year follow-up, resulting in a comprehensive study design. As there are several scales that need to be filled out accurately by the participants and their parents, collecting and obtaining study data is time-consuming. As a result, recruiting and follow-up such a large sample of patients with NSSI at a single hospital for 1 year is challenging. Our hospital is one of the largest and most professional psychiatric hospitals in Southwest China. As a result, we were able to examine such important diseases in a large patient cohort.

This preliminary study has significantly contributed to our understanding of the potential effects of parent–adolescent family relationship cognition and cognitive differences on the treatment effect of NSSI. However, this study has some limitations. First, all data were obtained using self-completed questionnaires, with some degree of reporting bias. Second, parent–adolescent family cognitive data were only collected at baseline, failing to assess changes in their cognition of family relationships during treatment. It was best to evaluate their family relationship cognitive data after 1 year follow-up to explore the effects of improved NSSI in adolescents on family relationship cognition. Third, owing to the wide gap between rich and poor among regions in China, NSSI-related variables, such as economic conditions or regions, were limited. To further understand the potential effects of parent–adolescent family relationship cognition and cognitive differences on NSSI in China, a large-scale study involving more regions and populations conducted in multiple centres is required.

## 5. Conclusion

There were certain differences in the cognition of family relationships between parents and adolescents, and subjective family relationship cognition and cognitive differences had a significant effect on the treatment effect of NSSI in adolescents. Helping them identify the cause of cognitive differences and conducting systematic family therapy from the points of difference may be another perspective to improve the treatment effect of NSSI in adolescents.

## Data availability statement

The raw data supporting the conclusions of this article will be made available by the authors, without undue reservation.

## Ethics statement

This study was approved by the Ethics Committee of the Fourth People’s Hospital of Chengdu (No. 96 201830). Written informed consent was obtained from all participants and their parents or guardians.

## Author contributions

YalL and XL designed the research protocol, analysed the data, and drafted the manuscript. ND conducted the study and provided the funding resources. YuL, YX, CL, JC, YaoL, LL, DS, JJ, HC, and TL critically revised the manuscript. All authors contributed to the article and approved the submitted version.

## Funding

Financial support of this work was provided by Sichuan Medical Association (Q20017), Chengdu Municipal Health Commission (2021103 and 2022441), and Sichuan Provincial Department of Science and Technology (2023YFS0219 and 2023YFS0228). The funding agencies did not have any role in the design of the study, collection, analysis, and interpretation of data, and in writing the manuscript.

## Conflict of interest

The authors declare that the research was conducted in the absence of any commercial or financial relationships that could be construed as a potential conflict of interest.

## Publisher’s note

All claims expressed in this article are solely those of the authors and do not necessarily represent those of their affiliated organizations, or those of the publisher, the editors and the reviewers. Any product that may be evaluated in this article, or claim that may be made by its manufacturer, is not guaranteed or endorsed by the publisher.

## Supplementary material

The Supplementary material for this article can be found online at: https://www.frontiersin.org/articles/10.3389/fpsyt.2023.1183916/full#supplementary-material
