# Supplementary material for: The effects of family environment cognition and its difference perceived by adolescents and their parents on the treatment effect of non-suicidal self-injury behaviors in adolescents: a 1-year prospective cohort study
Source: Front Psychiatry. 2023 Sep 12;14:1183916. doi: 10.3389/fpsyt.2023.1183916 (PMC10523313; doi:10.3389/fpsyt.2023.1183916)
Supplement: Supplementary file 1 [file Data_Sheet_1.docx]

# Questionnaires for the LoPDS cohort study

# Participant overview

Participant ID:

Center:

Registration date:

Recruitment date:

## Recruitment

### 1. Registeration

* Participant ID： * Participant’s name： *MW Contact Date： *Study ID：

### 2. Agreement

*I agree to take part in this study. Yes NO

1. I have been reading the informed consent, the researchers have explained the purpose, contents, risks and benefits of this research to me clearly. My questions so far have been answered. I understand the information printed on this form, and I volunteered for this study.

Yes NO

1. I agree to donate my biological samples (including blood, urine, hair, nails, buccal smear) for this research. I know our biological samples such as blood, urine and hair could be sent to study abroad, and I know the donation is voluntary. I could withdraw from the study whenever I decide, which won’t affect the normal care. Yes NO

3. I agree the researchers to check on my medical records and personal information related to medicine. I know that my personal information will be kept secret. Yes NO

4. I agreed to the use of biological samples and personal information I donated in current and future scientific research (including commercial research and scientific research not related to this project). I know clearly that if this research could lead to new treatments and inventions of medical testing, I would not be able to gain commercial benefits from it.

Yes NO

5. I have the opportunity to invite my family or friends to help me to ask questions about this study, I know the person I should contact with if there is a problem. Yes NO

### 3. Enrolment

Group： ①NSSI ②Suicide

Inclusion Criteria：* NSSI

* 12-18 years old

***** Able to provide written, informed consent

Exclusion Criteria: ***** did not complete standardized questionnaires assessment

***** unable to participate in follow-up

### 4. Basic information

*name：

*native place：

*Telephone number：

*address：

* Patient ID：

*education：1- primary school 2- high school 3- University 4- university or above 5- illiterate 6- other:

*occupation：

*blood type：

*height： cm

*smoke（1-no 2- smoking before 3-still smoking, /d 4-History of passive smoking, years） *alcohol（1-no 2- drinking before 3-still drinking, g/week）

### 5. Past medical history

*heart disease：①yes ②no

*high blood pressure：①yes ②no

*nephritis：①yes ②no

*hepatitis：①yes ②no

*Tuberculosis：①yes ②no

* diabetes mellitus：①yes ②no

*blood disease：①yes ②no

* Mental illness：①yes (add type) ②no

*epilepsy：①yes ②no

*thyroid dysfunction：①yes ②no

*allergic history**：**①yes ②no

* operation history：①yes（add operation name and time） ②no

other：

### 6. Family History

*neuropathy：①yes（add anyone）②no

*dementia：①yes（add anyone）②no

*malformation：①yes（add anyone）②no

*genetic disease：①yes（add anyone）②no

*high blood pressure：①yes（mother or father） ②no

* diabetes mellitus：①yes（mother or father） ②no

*Cardiovascular diseases：①yes（mother or father） ②no

* Immune diseases：①yes（mother or father） ②no

*tumor history：①yes（mother or father） ②no

other：
