# Supplementary material for: The effects of family environment cognition and its difference perceived by adolescents and their parents on the treatment effect of non-suicidal self-injury behaviors in adolescents: a 1-year prospective cohort study
Source: Front Psychiatry. 2023 Sep 12;14:1183916. doi: 10.3389/fpsyt.2023.1183916 (PMC10523313; doi:10.3389/fpsyt.2023.1183916)
Supplement: Supplementary file 2 [file Data_Sheet_2.doc]

**Family Environment Scale-Chinese Version (****FES-CV)**

The use of the questionnaire requires that the subjects have a primary education or above. The main examiner should monitor the whole process of the subjects' completion of the scale. If the subjects cannot understand multiple items, they should stop the test and confirm that the answers are invalid.

This questionnaire is used to find out what you think of your family. Please confirm whether the following questions are in line with the actual situation of your family. If you think a question is in line with the actual situation of your family, please answer "Yes", if it does not or basically does not meet, please answer "No". If it is difficult to judge, you should answer according to the behavior of most family members or the situation that occurs frequently. If you are still not sure, answer according to your own estimates. Be sure to answer each question. Some questions with "★" means that the sentence has a negative meaning, please pay attention to correctly understand the content of the sentence. Remember, when this questionnaire refers to "family" you mean the small family that you share your food and lodging with. When answering the questionnaire, don't speculate on other people's views on your family, please answer according to the actual situation. Please write the answer in ( ).

**Participant ID:**

**Center:**

**Registration date:**

**Recruitment date:**

**Note: In this questionnaire, 1 means "yes" and 2 means "no". Please answer with 1 or 2.**

1 ( ) Our family members always give each other the greatest help and support.

2 ( ) Family members always keep their feelings in their hearts and do not disclose them to other family members.

3 ( ) Often quarrels at home.

4 ( ) ★ At home, we seldom act alone.

5 ( ) Family members do their best whatever they do.

6. Our family often talks about political and social issues.

7. Most weekends and evenings are spent at home by family members, instead of going out to participate in social and entertainment activities.

8 ( ) We all believe that no matter how difficult it is, children should first meet the needs of the elderly.

9 ( ) Major activities at home are carefully arranged.

10 ( ) ★ Family members rarely force other family members to abide by family rules.

We are bored at home.

12 ( ) We can say whatever we want at home.

13 ( ) ★ Family members seldom get angry with each other publicly.

14 ( ) We all encourage our family members to be independent.

15 ( ) In order to have a good future, family members spend almost all their energy.

16 ( ) ★ We seldom go out to listen to lectures, watch movies, or go to museums and exhibitions.

17 ( ) Family members often go out to friends' homes to play and eat together.

18 ( ) All family members believe that everything should conform to social customs.

19 ( ) Generally speaking, we all pay attention to keeping our homes in order.

20 ( ) ★ There are few fixed life rules and family rules at home.

21 ( ) Family members are willing to spend a lot of energy on family affairs.

22 ( ) Complaining at home can easily annoy family members.

23 ( ) Sometimes family members throw things when they are angry.

24 ( ) Family members think independently.

25 ( ) All family members believe that improving living standards is more important than anything else.

26 ( ) We all believe that learning new knowledge is more important than anything else.

27 ( ) ★ No one at home participates in various sports activities.

28 ( ) Family members often help the elderly and disabled around them in life.

29 ( ) In our family, when we need something, we often can't find it.

30 ( ) The time of eating and sleeping in our house is the same.

31 ( ) There is a harmonious atmosphere in our home.

32 ( ) Everyone in the family can tell their difficulties and troubles.

33 ( ) ★ Family members seldom lose their temper.

34 ( ) Everyone in our family is completely free to go in and out.

35 ( ) We all believe that competition is a good thing under any circumstances.

36 ( ) ★ We are not so interested in cultural activities.

37 ( ) We often watch movies or sports competitions, go out for outings, etc.

38 ( ) We believe that bribery is an acceptable phenomenon.

39 ( ) In our family, we attach great importance to punctuality.

40 ( ) Our family has a fixed way to do everything.

41 ( ) ★ Few people volunteer to do something at home.

42 ( ) Family members often express their feelings openly.

43 ( ) Family members often blame and criticize each other.

44 ( ) ★ Family members seldom consider the opinions of other family members when doing things.

45 ( ) We always reflect on ourselves and force ourselves to do things better each time.

46 ( ) ★ We seldom discuss issues related to scientific and technological knowledge.

47 ( ) Everyone in our family is particularly interested in one or two recreational activities.

48 ( ) We believe that no matter what, the younger generation should accept their elders' advice.

49 ( ) People in our family often change their plans.

50 ( ) Our family attaches great importance to abiding by the fixed life rules and family rules.

51 ( ) Family members always support each other heartily.

52 ( ) If you express your dissatisfaction with family affairs at home, some people will feel uncomfortable.

53 ( ) Family members sometimes fight with each other.

54 ( ) Family members rely on their families' help to solve their difficulties.

55 ( ) ★ Family members don't care much about job promotion, academic performance and other issues.

56 ( ) Someone at home plays musical instruments.

57 ( ) ★ Except for work and study, family members do not often engage in recreational activities.

58 ( ) Family members voluntarily maintain public environmental sanitation.

59 ( ) Family members carefully keep their rooms clean and tidy.

60 ( ) Family members can go out at night without consulting with their families in advance.

61 ( ) ★ Our family has little collective spirit.

62 ( ) We can openly talk about our family's economic problems.

63 ( ) When family members have different opinions, we always avoid it to maintain harmony.

64 ( ) Family members hope that their families can solve problems independently.

65 ( ) ★ Our family members are not so positive about getting achievements.

66 ( ) Family members often go to the library.

67 ( ) Family members sometimes participate in recreational learning according to their personal hobbies or interests.

68 ( ) All family members believe that they should stick to the moral dogma.

69 ( ) The division of labor in our family is clear.

70 ( ) ★ There are no strict rules to constrain us in our family.

71 ( ) Family members always get along with each other.

72 ( ) Family members are careful not to hurt each other's feelings when speaking.

73 ( ) Family members often try to outdo each other.

74 ( ) If family members often act alone, it will hurt the feelings of others in the family.

75 ( ) It is an old habit of our family to work first and enjoy later.

76 ( ) Watching TV is more important than reading in our home.

77 ( ) Family members often participate in social activities outside the family in their spare time.

78 ( ) We believe that divorce is immoral no matter what.

79 ( ) ★ Our family has no plan to spend money.

80 ( ) The life rules or rules of our family cannot be changed.

81 ( ) Every member of the family has always received full attention.

82 ( ) Our family often talks about their sensitive issues spontaneously.

83 ( ) When family members have conflicts, they sometimes quarrel loudly.

84 ( ) In our family, we really encourage members to move freely.

85 ( ) Family members often compare with others to see who is good at study and work.

86 ( ) Family members like music, art and literature very much.

87 ( ) Our way of entertainment is watching TV and listening to the radio rather than going out.

88 ( ) We believe that improving the living standard at home is more important than strictly abiding by moral standards.

89 ( ) Someone must wash the dishes immediately after our meal.

90 ( ) Those who violate family rules at home will be severely criticized.
