# Supplementary material for: The effects of family environment cognition and its difference perceived by adolescents and their parents on the treatment effect of non-suicidal self-injury behaviors in adolescents: a 1-year prospective cohort study
Source: Front Psychiatry. 2023 Sep 12;14:1183916. doi: 10.3389/fpsyt.2023.1183916 (PMC10523313; doi:10.3389/fpsyt.2023.1183916)
Supplement: Supplementary file 3 [file Data_Sheet_3.docx]

**Non-suicide self-injury behavior evaluation questionnaire for adolescents**

1. **In the recent year, have you committed any of the following acts of intentionally hurting yourself, which are not intended to commit suicide, but may lead to bleeding, bruise or pain (actions taken to refresh yourself when tired are not included), according to the actual situation, type the corresponding answer ✔**

Entries No Occasionally Sometimes Often Always

1 Intentionally strangling yourself

2 Intentionally scratching yourself

3 Intentionally hitting your head against a hard object (e.g. wall, tree, etc.)

4 Intentionally hitting hard objects such as walls, tables, windows, floors, etc. with your fists

5 Intentionally injuring yourself with a fist, slap, or hard object

6 Intentionally pricking or stabbing yourself (e.g., with needles, staples, nibs, etc.)

7 Intentionally cutting yourself (e.g., with a blade, glass, etc.)

8 Intentionally biting yourself

9 Deliberately pulling off one's own hair

10 Intentionally burning or scalding oneself (e.g. with cigarette butts, boiling water, lighters or matches, etc.)

11 Deliberately rubbing the skin with something to make it bleed or bruised

12 Intentional engraving or symbols on the skin (excluding tattoos)

1. **If you have committed the above behaviors, what are the reasons for hurting yourself? Fill in according to the actual situation.**

Completely inconformity, inconformity, Uncertain, compliant, fully compliant

1 Express your anger

2 In order to avoid things that you don't like or make you unhappy

(such as avoiding school, doing homework or labor, etc.)

3 Make yourself feel not lonely

Relieve stress or anxiety

5 Ways of self-punishment or atonement

6. It can bring happiness, enjoyment and make you feel good

7 Control yourself and calm yourself down

8 Attract others' attention

9 Revenge others

Only in this way can we not hurt others

11 Protect yourself from others' attacks

12 Help yourself stop bad thoughts or thoughts

13 Friends have done this before

14 Have the desire to hurt yourself and can't stop

15 Get others' understanding

16 Dealing with sadness and disappointment

17 Show your despair and helplessness

18 Let others make changes

19 Escape from numbness and illusion

**3、 In the past two weeks, if the intensity of your idea of self-injury is graded from 0 to 10 (0 is no self-injury idea, 10 is self-injury idea at any time), you think you are:**

Level 0, 1, 2, 3, 4, 5, 6, 7, 8, 9, 10

**4、In the past 2 months, whether you have self-injury behavior: 1. Yes, 2. No, if you answer "Yes", please fill in the following table:**

Items not occasionally sometimes always

1 intentionally pinching oneself

2 Intentionally scratch yourself

3 deliberately hit hard objects (such as walls, trees, etc.) with your head

4 Intentionally hit hard objects such as walls, tables, windows and floors with fists

5 Intentionally hit yourself with fists, palms or hard objects

6 Intentionally prick or stab yourself (such as using needles, staples, pen tips, etc.)

7 Intentionally cut yourself (such as using a blade, glass, etc.)

8 Intentionally bite yourself

9 Pull off your hair intentionally

10 Intentionally burn or scald yourself (such as using cigarette butts, boiling water, lighters or matches, etc.)

11 Use something to deliberately rub the skin to make it bleed or congestion

12 Intentionally engrave words or symbols on the skin (excluding tattoos)

**5、 Have you ever had the impulse to hurt yourself (not implemented) in the last 2 weeks?**

1. Yes; 2. No; 3. If the answer is "Yes", please fill in the following table:

Frequency of self-injury impulse: single choice

1. Once in two weeks

2. Once a week

3. Twice a week

4. More than 3 times a week

5. More than 5 times a week

6. Once a day on average

8. More than 2 times a day on average
